# Migration and Women's Health Research (2000−2023): A bibliometric analysis of trends and gaps

**DOI:** 10.1016/j.dialog.2025.100210

**Published:** 2025-03-04

**Authors:** Aasif Hussain Sheikh, Snober Hamid, Bilal Ahmad Lone, Farheena Muzaffar, Manzoor Hussain

**Affiliations:** Department of Sociology, University of Kashmir, India

**Keywords:** Women, Migration, Health, Bibliometric analysis, Publication trends

## Abstract

This bibliometric study examines the scholarly landscape of migration and women's health, analyzing 1314 Scopus-indexed articles from 462 journals published between 2000 and 2023. Findings indicate a consistent increase in research output, reflecting growing global interest in this interdisciplinary field. Geographically, high-income countries (HICs), including the United States, Canada, the United Kingdom, and Australia, dominate contributions, while low- and middle-income countries (LMICs) remain underrepresented despite hosting significant migrant populations. International collaborations play a crucial role, with key institutions such as the University of California and the London School of Hygiene and Tropical Medicine shaping research efforts. The keyword co-occurrence analysis highlights migration, gender dynamics, mental health, and reproductive health as dominant themes. Persistent gaps in mental and reproductive healthcare access for migrant women emphasize the need for trauma-informed care (TIC), mobile bilingual healthcare services, and inclusive health policies. Disparities in research funding further exacerbate global health inequities, underscoring the necessity of equitable redistribution of resources, including redirecting at least 10 % of HIC research grants to LMIC-led studies. The COVID-19 pandemic magnified pre-existing vulnerabilities, stressing the importance of multilateral collaborations and sustainable policy interventions to enhance migrant healthcare access. This study provides valuable insights into research trends, collaboration networks, and thematic focus areas, offering a foundation for future interdisciplinary research and evidence-based policymaking aimed at promoting health equity for migrant women globally.

## 1 Introduction

A migrant is a person who relocates from their typical place of residence, domestically or internationally, either temporarily or permanently, and for multiple reasons” [1]. The increasing scale of global migration, with over 272 million international migrants documented in 2019, among them, nearly half are female migrants, accounting for 48.1 % of the global migrant population [2]., has made women's health a critical area of study and policy intervention [3]. A significant proportion of these migrants originate from low- and middle-income countries (World Bank). Migration is often influenced by a complex interplay of economic, social, familial, and political factors, and over the past 50 years, the intersection of women's migration and health has emerged as a significant field of research, reflecting the evolving dynamics of global mobility and gendered health experiences.

Migration is now recognized as a key determinant of health, positioning it as a fundamental issue on the global health agenda [4]. The health impacts of migration can be both positive and negative, depending on migratory conditions, experiences, and health policies in host countries. Within this broader framework, migrant women's health has gained increasing attention due to the unique challenges they face before, during, and after migration [5]. Compared to men, migrant women are more vulnerable to health deterioration, as they often encounter gender-specific challenges, including limited knowledge of contraception and STIs, restricted access to healthcare due to isolation and stigma, and experiences of violence and unintended pregnancies [6,7].

In the context of globalization, urbanization, and demographic shifts, women's migration has become increasingly diverse, encompassing work-related relocation, family reunification, forced displacement, and other mobility patterns (United Nations). These diverse migration trajectories intersect with multiple dimensions of women's health, including maternal and child health, reproductive health, mental health, communicable diseases, and access to healthcare services [8]. Among these, reproductive health needs demand urgent attention. Despite the structured environments of refugee and migrant settlements, women's reproductive health concerns are frequently overlooked and inadequately addressed, with health professionals often failing to cater to their specific needs. For instance, research on Haitian immigrant women has shown how cultural context and healthcare service provision significantly shape their experiences of intimate partner violence (IPV) [9]. Additionally, cultural norms, legal status, and service accessibility influence vulnerability levels and the ability of migrant women to seek support.

Migration from low-income to high-income countries can present health benefits for women, provided they are employed and fluent in the local language. Access to preventive screenings, such as cervical and breast cancer checkups, can improve their overall health, whereas such services may be unavailable in their home countries. Additionally, migration from rural to urban areas is associated with better health outcomes due to improved medical infrastructure and healthcare access [10]. However, migrant women who lack language skills or employment often struggle to navigate healthcare systems in host countries, limiting their ability to access essential health services [11]. Abhishek and Kannuri [12] highlight the significant healthcare barriers faced by migrants, especially those resettled on the peripheries of urban centers, where public health services remain inadequate. This problem is exacerbated for vulnerable groups, including women, the elderly, and individuals with disabilities.

The effects of migration on women's health are multifaceted, with distinct challenges and inequalities emerging throughout the migration process. Many migrant women experience emotional and economic stress, as well as familial separation and cultural discord, which can have adverse physical and mental health outcomes [13]. Understanding these complex health dynamics is essential for designing targeted health interventions and policies that address their specific needs. Historically, migration studies have focused predominantly on male experiences, often overlooking women's roles in migration patterns. However, in recent years, there has been growing recognition of feminized migration, wherein women migrate independently for economic reasons and increasingly serve as primary breadwinners, particularly in Southeast Asia and Latin America [14,15]. This shift has prompted greater scrutiny of the structural barriers that impact migrant women's health, including restricted access to healthcare facilities, which contribute to widening health disparities within migrant populations [16].

A previous bibliometric analysis examined global migration health research [17]. However, it did not specifically analyse women's health in the context of migration. Despite the growing number of female migrants over the past two decades and increasing awareness of their unique healthcare needs, no comprehensive assessment has been conducted to map and examine the scientific literature on migrant women's health. Therefore, there is an urgent need to prioritize women's health in all migration-related contexts. At the health policy level, greater attention must be given to protecting the rights of migrant and refugee women while acknowledging and supporting their contributions to health and social development.

This study aims to trace the trajectory of research on women's migration and health while identifying key trends, themes, and research gaps in the field. The analysis focuses on publication growth, geographical distribution, authorship trends, highly cited studies, international collaborations, key thematic areas, and leading journals that have contributed to the discourse on migration and women's health.

## 2 Methodology

The study utilized a bibliometric analysis to explore the intersection of migration and women's health by examining research articles published in Scopus-indexed journals. In bibliometric research, documents are extracted from a single database and analyzed using quantitative and qualitative methods [18,19]. Commonly used databases for such analyses include Scopus and Web of Knowledge. In this study, Scopus, an Elsevier database, was selected to retrieve publications on migration and women's health, encompassing research on migrant women's health outcomes. Scopus was chosen over other databases such as Google Scholar Web of Science, and Medline, due to its several advantages, including extensive indexing, citation tracking, and bibliometric analysis tools ([20]; [21,22]). One of its key strengths is the user-friendly access to bibliometric indicators, which facilitates efficient analysis of publication trends and impact. Additionally, since Medline is fully integrated within Scopus, it ensures comprehensive coverage of medical and health-related literature. This study focused exclusively on original peer-reviewed journal articles, excluding gray literature, conference papers, books, and book chapters. The study period was set from 2000 to 2023 to provide a two-decade perspective on research trends in migration and women's health.

### 2.1 Search strategy

To ensure methodological thoroughness, a structured keyword selection process was applied. The primary search terms—”migration,” “women,” and “health”—were used in titles, abstracts, and keywords. To account for regional terminology variations, a combination of Boolean operators (AND/OR), synonyms, and related terms was employed. Additional keywords were identified through preliminary literature reviews and expert consultations. This ensured that the selected articles are directly relevant to the intersection of these topics. To capture relevant variations, regional synonyms and alternative terminologies were incorporated. For example, “immigrant,” “refugee,” “asylum seeker,” and “displaced person” were included alongside “migration.” Similarly, health-related terms encompassed “maternal health,” “reproductive health,” “mental health,” “access to healthcare,” and “disease prevention.” These variations were cross-referenced with regional terminology differences, ensuring the inclusion of diverse linguistic representations.

To enhance methodological transparency, a PRISMA-style flow diagram was used to document the article selection process, outlining the number of initial records retrieved, screened, included, and excluded at each stage.

**Figure:**
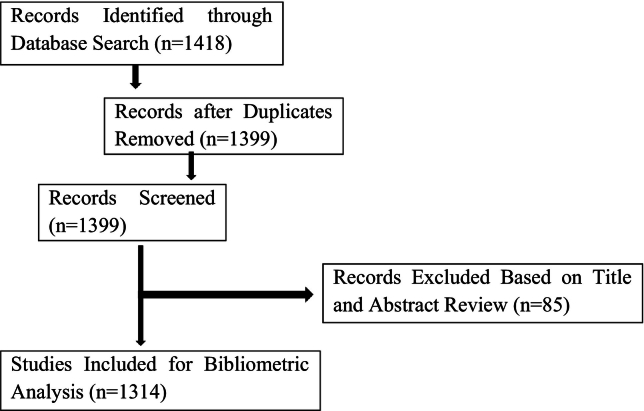

Inclusion criteria:

1. Studies published between 2000 and 2023 to provide a comprehensive two-decade overview.
2. Articles explicitly addressing both migration and women's health.
3. To capture social, behavioral perspectives and policy perspectives, the search was restricted to specific subject areas, including Sociology (SOCI), Arts and Humanities (ARTS), Psychology (PSYC), and Multidisciplinary fields (MULT).

Exclusion criteria:

1. Articles not directly related to both migration and women's health.
2. Studies lacking empirical data or bibliometric indicators.
3. Non-English language publications due to database indexing constraints.
4. Conference papers, reviews, books and book chapters and gray literature, ensuring focus on peer-reviewed journal articles.

### 2.2 Data analysis

The data was checked for duplicates using Zotero 6 for Windows software. Given Scopus's extensive coverage and citation data, the data collection spanned from January 2000 to December 2023, totaling 1314 documents sourced from 462 journals (Table 1). The average citation count per document was 20.9, with a cumulative reference count of 61,926 across all papers. Analysis revealed 3953 distinct author keywords and identified 4273 unique authors, with 248 documents being single-authored. On average, each document had 3.72 co-authors, with 25.27 % featuring international collaborations (Table 1). The study's subsequent analysis employed various bibliometric techniques, including descriptive statistics, network analysis, citation analysis, and temporal analysis, to identify patterns, trends, and influential publications in the field.

**Table 1:** Main information about data.

Description	Results
Timespan	2000:2023
Sources (Journals)	462
Documents	1314
Annual Growth Rate %	9.26
Document Average Age	8.68
Average citations per doc	20.9
References	61,926
*DOCUMENT CONTENTS*	
Keywords Plus (ID)	3953
Author's Keywords (DE)	2782
Authors	4273
Authors of single-authored docs	248
*Single-authored docs*	270
Co-Authors per Doc	3.72
International co-authorships %	25.27

### 2.3 Software tools used

In this study, a range of software tools were employed to conduct the bibliometric analysis. The primary tool utilized was VOSviewer (version 1.6.18), renowned for its capability to visualize various bibliographic data types on user-friendly maps. These maps include representations of co-authorship, citation patterns and keyword co-occurrence, all derived from bibliographic data. Additionally, Biblioshiny, an open-source software operating within RStudio (version 2022.02.2), was employed. Biblioshiny harnesses the functionalities of the bibliometrix package to facilitate scientific mapping analysis, aiding in the exploration and visualization of relationships and patterns within bibliographic data.

Furthermore, MS Access, MS Excel and Origin Pro (2023) served as indispensable tools for data analysis, offering robust capabilities for organizing and processing bibliographic data. One notable analysis conducted using Biblioshiny was the generation of a three-field plot, illustrating the interplay among authors, countries, and sources. This plot provides insights into collaborations, geographic distribution, and publication sources within the field under scrutiny.

### 2.4 Ethical approval

This study utilized publicly available bibliometric data from the Scopus database and did not involve human participants or sensitive personal information. As such, no ethical approval was required.

## 3 Results and discussion

### 3.1 Yearly trends in the production of articles in the field of migration and women's health

**Fig. 1:**
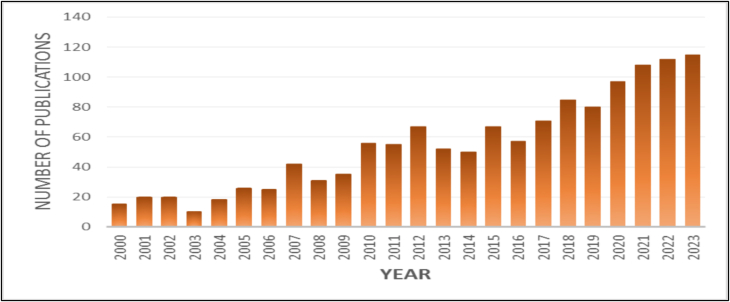
Year wise publication trend concerning migration and women's health.

The annual publication trend of research articles on migration and women's health from 2000 to 2023 exhibits a consistent upward trajectory, highlighting a growing scholarly focus on the subject (Fig. 1). The data reveal fluctuations in publication output, indicating periods of both acceleration and deceleration in research activity. Notably, a decline in publications was observed in 2003, which may be attributed to shifting research priorities or external factors influencing funding and academic interest. However, from 2007 onward, there was a significant increase in publication volume, reflecting renewed interest and intensified academic engagement in addressing migration-related health concerns among women.

A substantial surge in research output was recorded in 2020 and 2021, which coincides with the COVID-19 pandemic. This spike suggests that the global health crisis amplified awareness of the vulnerabilities faced by migrant women, resulting in an increase in scholarly discourse and policy-driven research efforts. The peak of publication activity occurred in 2022 and 2023, with 112 and 115 articles published, respectively, representing the highest research output within the study period. This peak underscores the culmination of scholarly endeavors aimed at examining the multifaceted dimensions of migration and its impact on women's health.

### 3.2 Geographic distribution of migration and Women's Health Research publications

An analysis of the geographic distribution of research publications (Table 2) reveals that certain high-income countries dominate the field in terms of both quantity and impact. The United States emerges as the leading contributor, accounting for 35.78 % (498 articles) of total publications, with an average of 21.91 citations per article, indicating substantial scholarly engagement. Canada follows closely, contributing 26.72 % (372 articles), with the highest average citation count of 31.56, reflecting both a high research volume and significant impact. Similarly, the United Kingdom (24.64 %, 343 articles) and Australia (17.24 %, 240 articles) maintain high average citation rates of 32.20 and 22.06, respectively, highlighting their influential contributions to the field.

**Table 2:** Country wise trend of publications related to Migration and Women's Health.

Region	Number of Articles	Percentage of Articles	Total Citations	Average Article Citations
USA	498	35.78	7209	21.91
CANADA	372	26.72	3061	31.56
UK	343	24.64	2640	32.20
AUSTRALIA	240	17.24	1191	22.06
SWEDEN	173	12.43	721	15.67
CHINA	159	11.42	539	19.25
FRANCE	145	10.42	489	37.62
NETHERLANDS	135	9.70	473	20.57
SPAIN	114	8.19	407	15.65
INDIA	106	7.61	397	20.89
GERMANY	100	7.18	383	17.41
ITALY	94	6.75	372	16.91
SWITZERLAND	86	6.18	308	30.80
SOUTH AFRICA	78	5.60	305	25.42
BELGIUM	77	5.53	200	9.09
MEXICO	72	5.17	177	9.32
NORWAY	53	3.81	161	10.73
TURKEY	49	3.52	152	5.85
DENMARK	46	3.30	147	29.40
THAILAND	42	3.02	139	9.93
BRAZIL	41	2.95	130	43.33
KENYA	38	2.73	128	25.60
ISRAEL	31	2.23	99	7.62
COLOMBIA	27	1.94	94	31.33
NIGERIA	27	1.94	88	17.60

A particularly noteworthy finding is the case of France, which, despite producing a smaller number of publications (145 articles, 10.42 %), exhibits the highest average citation rate of 37.62. This suggests that French research in this domain has high academic influence despite lower publication frequency. Other countries, including the Netherlands, Spain, India, Germany, and Italy, also contribute significantly, adding to the global discourse on migration and women's health. Additionally, countries such as Switzerland, South Africa, Belgium, Mexico, and Norway, though publishing fewer articles, demonstrate meaningful contributions, reflecting the diversity of global research efforts in this field.

**Fig. 2:**
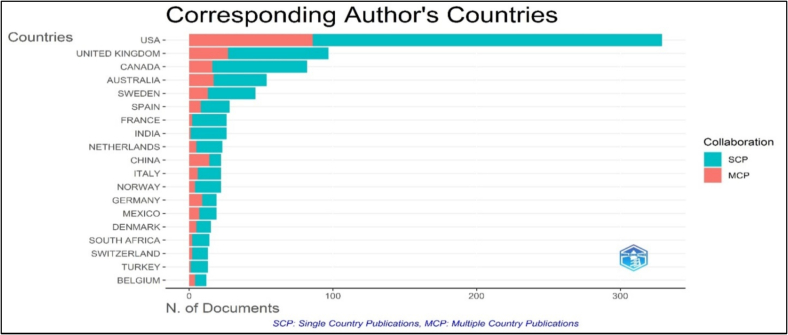
Distribution of corresponding authorship in publications related to Migration and Women's Health Research by Country.

Fig. 2 illustrates the corresponding authorship distribution by country, highlighting the dominance of certain nations in research output. The United States leads with 329 corresponding author articles comprising 243 single-country publications (SCPs) and 86 multiple-country publications (MCPs), yielding an MCP ratio of 0.261, indicating balanced involvement in both independent and collaborative research. Similarly, the United Kingdom (288 articles, 238 SCPs, 50 MCPs, MCP ratio of 0.174) demonstrates a strong presence in domestic research initiatives, while Canada (97 articles, 70 SCPs, 27 MCPs, MCP ratio of 0.278) exhibits higher collaborative engagement compared to other nations.

**Fig. 3:**
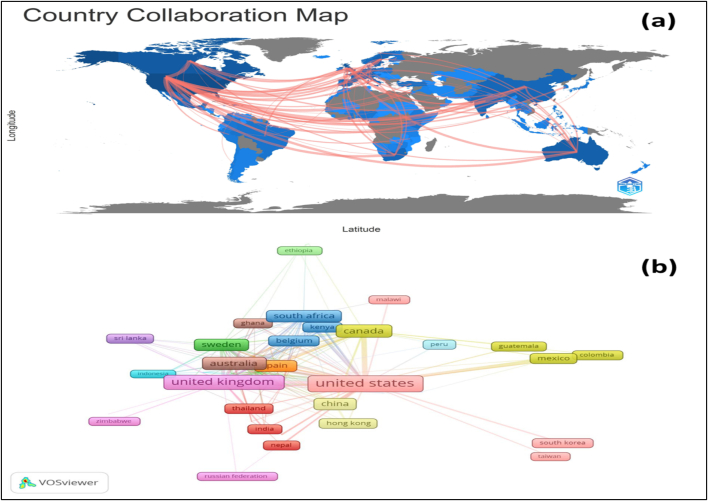
Map illustrating collaboration among countries, where red lines signify the network of collaboration among nations (a), network visualization, each country is represented by a node, with the color indicating the country's cluster. Lines connecting the nodes represent the links between countries, with the distance between two nodes indicating the intensity of their collaborative efforts (b). (For interpretation of the references to color in this figure legend, the reader is referred to the web version of this article.)

The country collaboration network (Fig. 3) further provides insights into international research partnerships by measuring Total Link Strength (TLS), an indicator of collaboration intensity between nations. The United States exhibits the highest TLS (224), suggesting extensive international collaboration, followed by the United Kingdom (TLS: 131) and Canada (TLS: 67). Other countries with notable collaboration strengths include Germany (TLS: 51) and Japan (TLS: 5). conversely, countries such as Argentina (TLS: 0), Benin (TLS: 3), and Vietnam (TLS: 6) exhibit limited international collaboration, indicating potential areas for expanding research partnerships.

### 3.3 Most relevant affiliations and authors shaping research in the field of migration and women's health

**Fig. 4:**
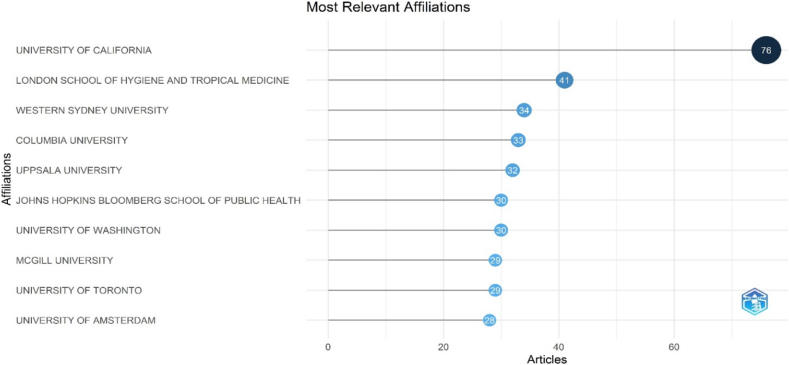
Organizations/ affiliations with highest number of publications in the field of migration and women's health.

An analysis of institutional contributions to migration and women's health research (Fig. 4) highlights the University of California as the most prolific institution, with 76 published articles. Other leading contributors include the London School of Hygiene and Tropical Medicine (41 articles), Western Sydney University [9], Columbia University [23], and Uppsala University [24]. Additionally, Johns Hopkins Bloomberg School of Public Health and the University of Washington each contributed 30 articles, further demonstrating the engagement of globally recognized institutions in this research domain.

**Fig. 5:**
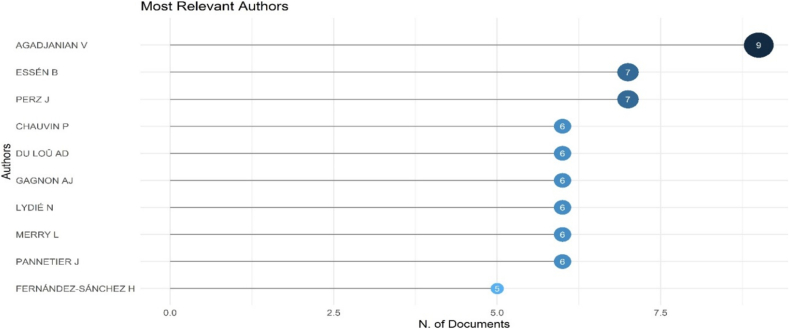
Most relevant authors who have made scientific contribution in the research area of migration and women's health.

**Fig. 6:**
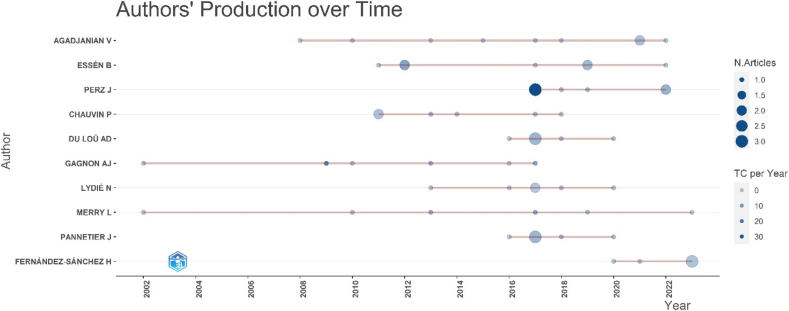
Article production of authors over time in the field of migration and women's health.

The most influential authors in this field are presented in Fig. 5 and Table 3. Agadjanian V emerges as the most prolific scholar, having authored 9 articles, with a fractionalized count of 3.92, reflecting significant collaborative contributions. Other prominent authors include Essén B and Perz J, each with 7 publications, indicating consistent scholarly engagement. (See Fig. 6.)

**Table 3:** Top 10 authors with highest impact in the field of migration and women's health.

Element	h_index	g_index	m_index	Total citations	Pub. Year_start
PERZ J	7	7	0.875	316	2017
AGADJANIAN V	6	8	0.353	70	2008
CHAUVIN P	6	6	0.429	153	2011
ESSÉN B	6	7	0.429	189	2011
GAGNON AJ	5	6	0.217	449	2002
MANDERSON L	5	5	0.208	130	2001
MERRY L	5	6	0.217	200	2002
USSHER JM	5	5	0.625	136	2017
GARCIA MA	4	5	0.444	71	2016
GOLDENBERG SM	4	5	0.364	106	2014

The academic impact of these authors is assessed through h-index, g-index, and m-index values (Table 3). Perz J exhibits an h-index of 7 and a g-index of 7, signifying high research impact, whereas Agadjanian V holds an h-index of 6 and a g-index of 8, suggesting enduring contributions within the field. Furthermore, authors such as Chauvin P, Du Loû AD, Gagnon AJ, and Merry L contribute substantially to the research landscape, enriching the multidisciplinary discourse on migration and women's health.

### 3.4 Co-occurrence of keywords

**Fig. 7:**
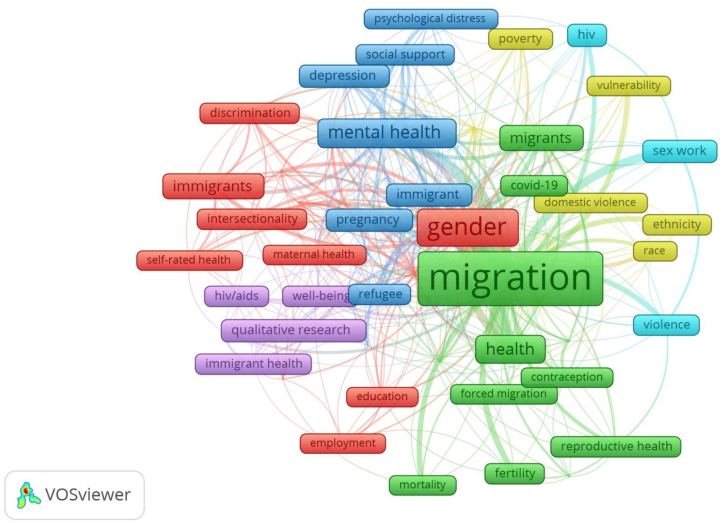
Network visualization, each keyword is represented by a node, with the color indicating the keyword cluster. Lines connecting the nodes represent the links between keywords, with the distance between two nodes indicating the intensity of their connections.

A comprehensive analysis into the prevalent themes and topics within the domain of migration and women's health research were analyzed. A co-occurrence analysis of keywords (Table 4, Fig. 7) provides insights into dominant research themes. “Migration” (255 occurrences, TLS: 256) is the most frequently appearing keyword, reinforcing its central role in this research domain. “Immigration” (133 occurrences, TLS: 152), “Gender” (126 occurrences, TLS: 150), and “Women” (68 occurrences, TLS: 87) also feature prominently, highlighting the focus on gendered migration experiences.

**Table 4:** To 20 most frequent keywords.

S. No	Keyword	Occurrences	total link strength
1	Migration	255	256
2	Immigration	133	152
3	Gender	126	150
4	Women	68	87
5	Mental Health	63	91
6	Health	55	73
7	HIV/AIDS	48	32
8	Refugees	46	64
9	Pregnancy	24	29
10	Depression	23	33
11	Internal Migration	22	22
12	Sex Work	20	23
13	Acculturation	19	30
14	Culture	19	23
15	Intersectionality	19	19
16	Intimate Partner Violence	17	15
17	Reproductive Health	17	17
18	Discrimination	16	25
19	Ethnicity	16	14
20	Covid-19	15	19

Mental health issues receive substantial attention, with “Mental Health” (63 occurrences), “Depression” [1], and “HIV/AIDS” [25] being frequently discussed topics. Additionally, reproductive health concerns, including “Pregnancy” (24 occurrences), “Reproductive Health” [26], and “Intimate Partner Violence” [26], underscore the vulnerabilities faced by migrant women. The emergence of COVID-19 (15 occurrences, TLS: 19) as a keyword reflects the impact of global health crises on migrant communities.

Migrant and refugee women constitute a highly vulnerable group to mental and reproductive health outcomes. Mental health has gained prominence in global health discussions, leading to initiatives like the WHO's Comprehensive Mental Health Action Plan 2013–2020, which emphasizes governance, care, and research [27]. The COVID-19 pandemic has further significantly impacted both the mental health and reproductive of migrant women, exacerbating pre-existing vulnerabilities and introducing new stressors. Many migrant women experienced heightened emotional, physical and mental strain due to increased caregiving demands, worsening financial situations, increased social isolation during the pandemic ([28,29]; & [30]). Women migrants reported a disproportionately negative impact on mental health compared to their male counterparts, with 86.9 % indicating adverse effects [28]. Similarly migrant women often encounter systemic barriers, such as restrictive immigration laws and policies that limit their access to healthcare services, including reproductive health care [31,32]. Women and girls migrating face heightened risks of gender-based violence and reproductive health issues, exacerbated by their precarious living conditions during migration [33]. Many experience violations of their sexual and reproductive rights, including limited access to contraception and safe abortion services [32,33]. Despite increased focus, substantial knowledge gaps remain, particularly regarding effective implementation in LMICs, indicating a disconnect between policy and practice ([34]; [27]). There is a disparity between research outputs and the priorities identified by stakeholders, particularly in low-income countries, where infectious diseases dominate research focus [35].

### 3.5 Health funding

Disparities in global health research funding play a critical role in shaping research priorities, data accessibility, and structural inequalities, particularly in the field of migration and women's health. Research funding remains highly concentrated in high-income countries (HICs), while low-income countries (LICs) receive only a fraction of global health research investments. A recent analysis revealed that LICs accounted for merely 0.2 % of all health research funding in 2020, underscoring the stark imbalance in financial allocations ([36]). This lack of funding severely restricts the research capacity and output of LICs, exacerbating global health inequities and perpetuating knowledge gaps on migrant women's health in underrepresented regions.

Many funding agencies prioritize research agendas aligned with HIC interests, often sidelining the health priorities of low- and middle-income countries (LMICs). The limited financial resources of LMIC governments further hinder investments in health research, resulting in an overreliance on external funding sources that may not fully address local health concerns [37]. A more equitable funding model necessitates greater collaboration with LMIC researchers, ensuring that funding mechanisms are responsive to regional health needs and that data-sharing practices are improved [38,39]. The availability of comprehensive health data is essential for developing evidence-based interventions, yet only a small percentage of research publications provide full access to their underlying datasets, limiting the ability to rapidly respond to health crises ([40]; [39]).

Funding disparities are particularly pronounced in women's health research, which remains significantly underfunded compared to other areas of global health. In 2020, only 5 % of global research and development (R&D) funding was allocated to women's health research, with 4 % designated for women's cancers and a mere 1 % for all other women-specific health conditions [41]. Additionally, 25 % of this funding was concentrated on fertility research, leaving critical areas such as maternal health, mental health, and gender-based violence largely neglected. This imbalance not only limits advancements in women's health research but also reinforces systemic gender inequities in healthcare policy and access.

The COVID-19 pandemic significantly reshaped global health research priorities, particularly concerning migration and women's health. The pandemic amplified awareness of the vulnerabilities of migrant women, many of whom serve as frontline workers while remaining marginalized in healthcare access [42]. The urgent need for pandemic-specific interventions led to a surge in scientific collaborations, with institutions forming new partnerships to address gender-related health concerns [43]. This crisis also highlighted the necessity for sex-disaggregated data and gender-specific health interventions, particularly for pregnant and lactating women, who faced unique health risks during the pandemic [44]. However, while the pandemic catalyzed important discussions on migrant women's health, there is a risk that long-standing systemic inequalities may continue to be overlooked in post-pandemic health agendas [45].

### 3.6 Covid-19 and migrant women

The Health Emergency and Disaster Risk Management (H-EDRM) framework, established in 2019, recognizes migrants and women as distinct groups that are at heightened risk and disproportionately affected by disasters relative to the general population ([46]). For women, vulnerability arises from the systematic differences in power relations and social hierarchies as well as gender roles, which influence their socioeconomic status and level of agency ([47,48]; & [26]). During the COVID-19 pandemic, women experienced worse socioeconomic impacts, faced access constraints to sexual and reproductive care [25], and bore the burden of the increased responsibilities for childcare. The underrepresentation of women in leadership roles during the pandemic limited the consideration of gender-specific needs in humanitarian responses. Women were largely excluded from national COVID-19 taskforces. Ultimately, responses to the pandemic have been gender-blind and inadequate given the disproportionate impacts of COVID-19 on women [49]. Women also faced disproportionate job losses leading to the feminization of poverty [50,51]. One of the key lessons learned from the COVID-19 pandemic is the importance of ensuring access to healthcare for vulnerable populations during crises incorporating strategies that prioritize equitable access to services for all demographics, particularly those who are marginalized or face systemic barriers. [42,25].Effective public policies that support women's employment and social coverage are crucial for mitigating these impacts in future crises [50].

## 4 Limitations and future research

While this study provides valuable insights into migration and women's health research, it has several limitations. Firstly, the analysis relied exclusively on the Scopus database, which, although comprehensive, does not include all relevant research. Non-Scopus-indexed literature, particularly studies published in regional and non-English-language journals, may have been overlooked, potentially limiting representation from low- and middle-income countries (LMICs) where migrant health challenges are often most severe. Future research should incorporate additional databases, such as Web of Science and PubMed, to ensure broader coverage. Secondly, the choice of bibliometric software tools, including VOSviewer and Biblioshiny, may have influenced the visualization of results. While these tools are widely used in bibliometric research, alternative analytical techniques, such as machine learning-driven text mining, could offer deeper insights into emerging research trends. Another limitation is the exclusion of non-English publications, which may have resulted in the omission of significant research findings from non-English-speaking regions. This restriction highlights the need for multi-lingual bibliometric studies to enhance the global representation of migrant women's health research. Despite efforts to ensure data accuracy, undetected duplicates may have influenced citation and co-authorship analyses, though their impact is expected to be minimal.

To address these limitations, future studies should focus on:

1. Maternal and Reproductive Health in LMICs – Research should explore barriers to maternal healthcare access, contraceptive use, and pregnancy-related complications in low-resource settings. Expanding mobile clinic services, particularly in peri-urban settlements and hilly remote areas, can improve reproductive healthcare access for migrant women [12,52,25].
2. Mental Health of Migrant Women – There is a critical need for studies on post-traumatic stress disorder (PTSD), depression, and anxiety among migrant women, particularly those displaced due to conflict or economic hardship. The effectiveness of trauma-informed care (TIC) in improving psychosocial outcomes warrants further investigation.
3. Funding Equity and Research Collaboration – Addressing global health funding disparities is essential. Redirecting 10 % of high-income country (HIC) research grants to LMIC-led initiatives could help strengthen local research capacity and ensure contextually relevant studies on migrant health issues [37].
4. Long-Term Impact of COVID-19 on Migrant Women – The pandemic disrupted healthcare services, employment, and social support systems for migrant women. Future research should examine how these disruptions have affected their long-term health and economic well-being.

## 5 Conclusion

This study employed a rigorous bibliometric approach, analyzing 1314 articles from 462 Scopus-indexed journals spanning 2000–2023. The findings demonstrated a steady upward trend in publication output, reflecting growing scholarly attention to the intersection of migration and women's health. However, geographical disparities persist, with research contributions concentrated in developed countries like the United States, Canada, the United Kingdom, and Australia, despite higher migrant populations in underrepresented regions. The imbalance in research focus carries significant ethical implications, as the health needs of migrant women in low-income countries remain overlooked. Funding disparities further exacerbate global health inequities, perpetuating regional research gaps. Developed nations secure the majority of global health research grants, leaving low- and middle-income countries (LMICs) with limited resources to conduct independent studies or implement policy-driven solutions. This imbalance not only influences research priorities but also restricts the generation of locally relevant health data, making it difficult to develop tailored healthcare models for migrant women in resource-poor settings. Addressing this disparity requires greater international collaboration, equitable funding distribution, and improved data-sharing initiatives to amplify research efforts in neglected regions. The frequent citation of mental health challenges in this research underscores the urgency of integrating trauma-informed care (TIC) within migrant healthcare services. Culturally competent approaches—such as training healthcare providers on migration-specific stressors, expanding bilingual health services, and deploying mobile clinics—can bridge accessibility gaps and improve outcomes for migrant women. Similarly, reproductive health disparities remain a pressing issue, with barriers to prenatal care, safe childbirth, and maternal support necessitating targeted interventions. Policymakers should collaborate with community organizations to bridge healthcare gaps and ensure accessible, inclusive services. The Covid-19 pandemic further exposed the vulnerabilities of migrant health systems, particularly in developing nations. It reinforced the moral imperative for high-income countries to provide funding and share health data, ensuring that crises do not deepen existing inequities. Achieving Sustainable Development Goal 3 (SDG-3)—ensuring healthy lives and well-being for all—requires a commitment to reducing systemic disparities, amplifying migrant women's voices, and embedding equity-driven strategies in global health policies.

## Funding

No funds, grants, or other support was received. The authors have no competing interests to declare that are relevant to the content of this article.

The data used in the study can be made available upon reasonable request.

## CRediT authorship contribution statement

**Aasif Hussain Sheikh:** Writing – original draft, Software, Methodology, Formal analysis, Data curation, Conceptualization. **Snober Hamid:** Validation. **Bilal Ahmad Lone:** Writing – review & editing. **Farheena Muzaffar:** Visualization, Investigation. **Manzoor Hussain:** Writing – review & editing, Supervision.

## Declaration of competing interest

The authors declare that they have no known competing financial interests or personal relationships that could have appeared to influence the work reported in this paper.
